# Cryo-laser scanning confocal microscopy of diffusible plant compounds

**DOI:** 10.1186/s13007-018-0356-x

**Published:** 2018-10-13

**Authors:** Kevin Vidot, Cédric Gaillard, Camille Rivard, René Siret, Marc Lahaye

**Affiliations:** 1 UR 1268 Biopolymères Interactions Assemblages, INRA, 44300 Nantes, France; 2 USC 1422 GRAPPE, INRA, Ecole Supérieure d’Agricultures, SFR 4207 QUASAV, 55 rue Rabelais, 49100 Angers, France; 3 Synchrotron SOLEIL, L’Orme des Merisiers, Saint-Aubin, 91192 Gif-Sur-Yvette Cedex, France; 4 UAR 1008 DPT CEPIA, INRA, 44300 Nantes, France

**Keywords:** Cryogenic fixation, Cryo-confocal microscopy, Metallic ions, Phenolics, Apple, Grape

## Abstract

**Background:**

The in vivo observation of diffusible components, such as ions and small phenolic compounds, remains a challenge in turgid plant organs. The analytical techniques used to localize such components in water-rich tissue with a large field of view are lacking. It remains an issue to limit compound diffusion during sample preparation and observation processes.

**Results:**

An experimental setup involving the infusion staining of plant tissue and the cryo-fixation and cryo-sectioning of tissue samples followed by fluorescence cryo-observation by laser scanning confocal microscopy (LSCM) was developed. This setup was successfully applied to investigate the structure of the apple fruit cortex and table grape berry and was shown to be relevant for localizing calcium, potassium and flavonoid compounds.

**Conclusion:**

The cryo-approach was well adapted and opens new opportunities for imaging other diffusible components in hydrated tissues.

## Background

Plant growth involves intricate relations between cell water compartmentalization and cell wall mechanical properties [1]. These relations involve cations for osmotic regulation and cell wall polysaccharide interactions, remodelling or deconstruction [2–7], but detailed knowledge on cation roles and interactions is impeded by their high mobility and/or low abundance in turgid tissue. Analytical methods for cation localization with a high spatial resolution are thus required. Due to the high water content of growing plant tissue, restraining the diffusion of mobile ions and preserving tissue integrity remain a challenge [8–10].

Specific chemical or physical fixation methods of plant tissue structures for microscopic observations exist [11]. Cryo-techniques coupled to cryo-observation in large fields of view using fluorescent techniques are particularly suited to localize metallic cations and diffusible compounds at low concentrations. The cryogenic fixation of plant tissues for light microscopy, called cryo-observation, has been described [12] but has been rarely used in the fluorescent mode [13]. This scarcity is most likely due to the difficulty in keeping the cold chain intact between sample cryo-fixation, cryo-sectioning and sample observation in frozen conditions. To that end, a method of fluorescence staining followed by cryo-fixation and cryo-observation by laser scanning confocal microscopy (LSCM) was developed and applied to the apple fruit cortex and table grape berry as models of turgid plant organs.

## Results

### Staining approaches

**Fig. 1:**
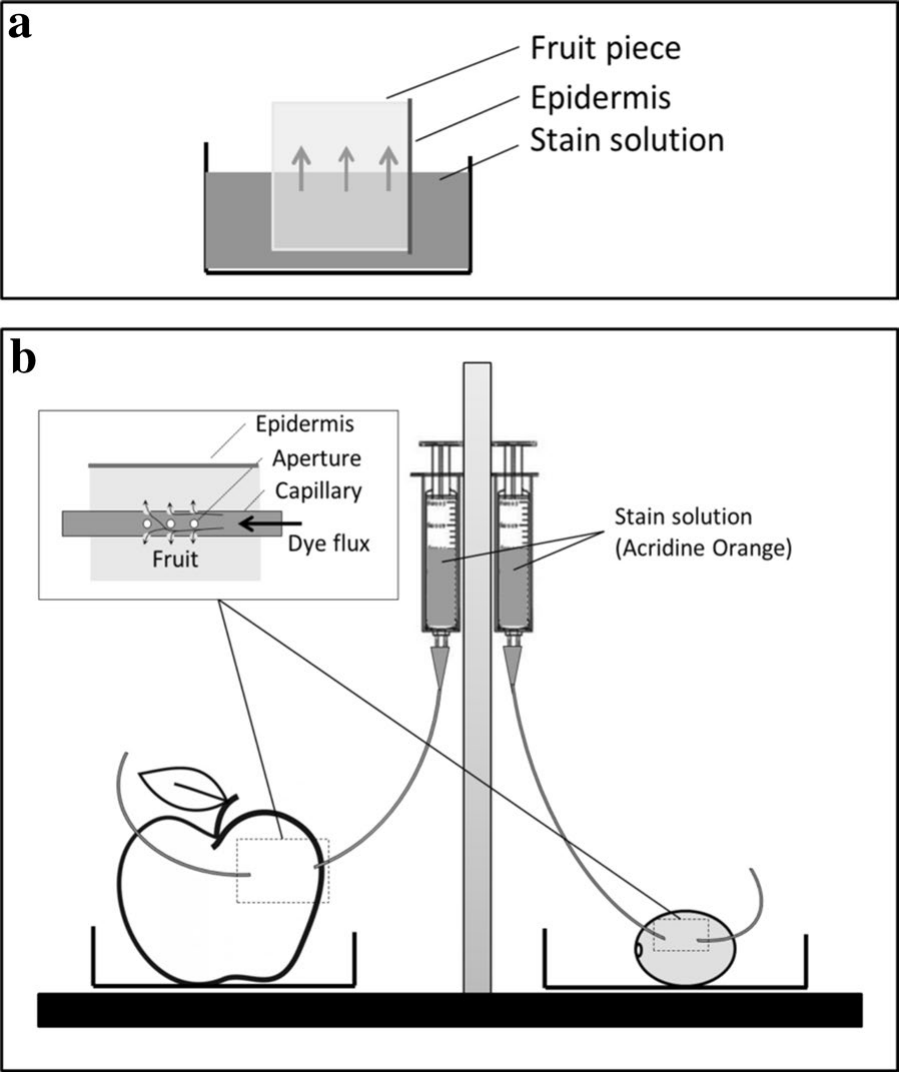
Staining methods by **a** infusion and **b** perfusion

Staining of the sample with aqueous dye solutions must be completed prior to cryo-fixation. In the present case, it was achieved by the infusion or perfusion (Fig. 1) of fresh samples using acridine orange (AO), a fluorescent dye for the cell walls and anionic sites of cell organelles, DNA and RNA [14–16]. Compared to the direct staining of fresh sample sections, AO infusion was efficient, while perfusion showed limited dye diffusion in the vicinity of the capillary and required an increase of the laser intensity to reveal probe fluorescence. Although less efficient, the latter method may be useful to study specific tissue locations with limited operation artefacts. Due to its efficiency and simplicity, infusion staining was chosen in the following study. Such a staining method that is used prior to fixation and cutting a section has already been reported to successfully stain potassium in leaves [17].

### Cryo-fixation

**Fig. 2:**
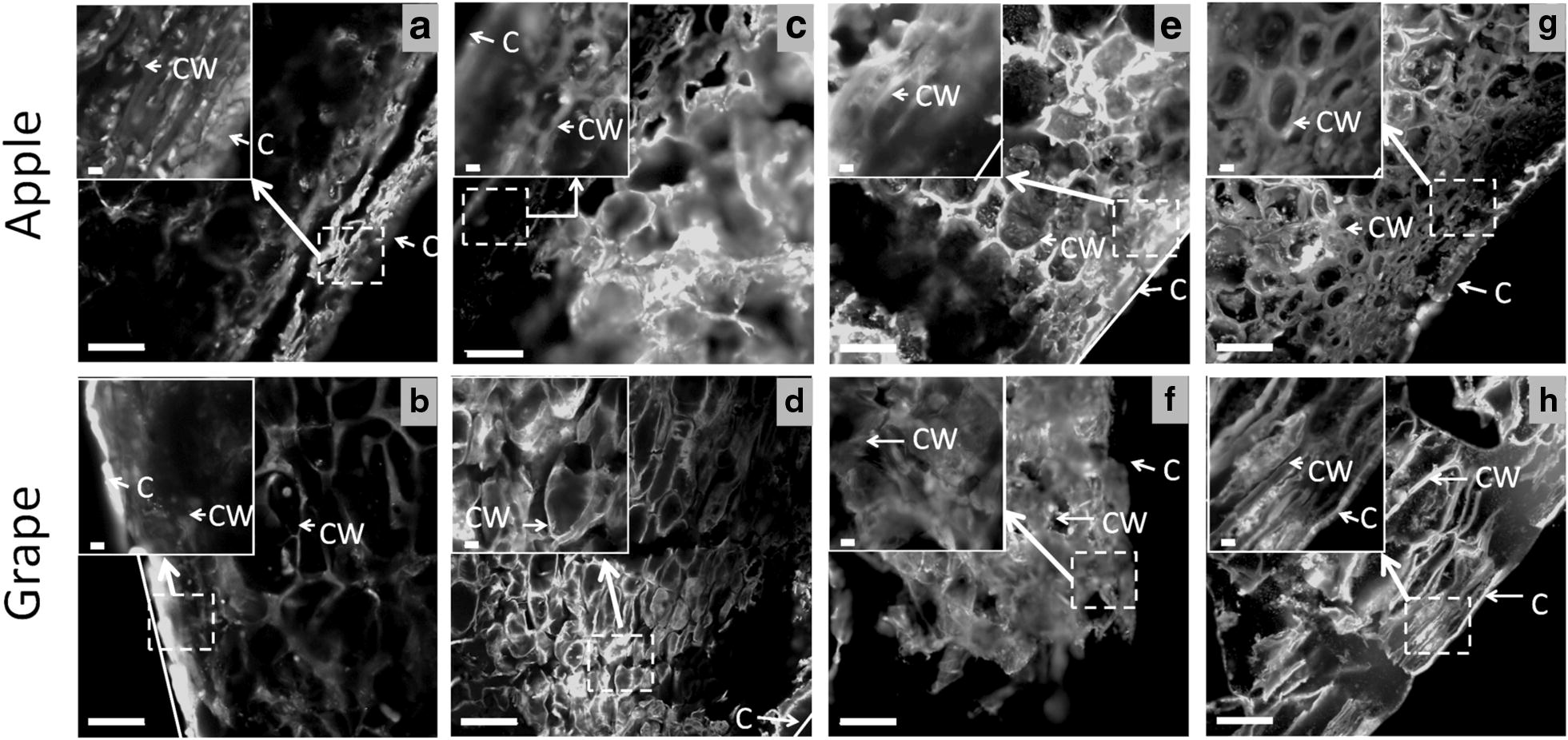
Effect of cryo-fixation methods on the apple cortex and grape berry tissue structure: **a**–**b** frozen samples at − 20 °C; **c**–**d** fast-frozen samples in liquid nitrogen; **e**–**f** fast-frozen samples in cooled isopentane; and **g**–**h** frozen samples at − 30 °C under microwaves. All sections were prepared from apples (**a**, **c**, **e**, **g**) or grapes (**b**, **d**, **f**, **h**) and stained by infusion with Acridine Orange. CW: Cell wall; C: Cuticle. Scale bar: 100 and 10 µm in inset

**Fig. 3:**
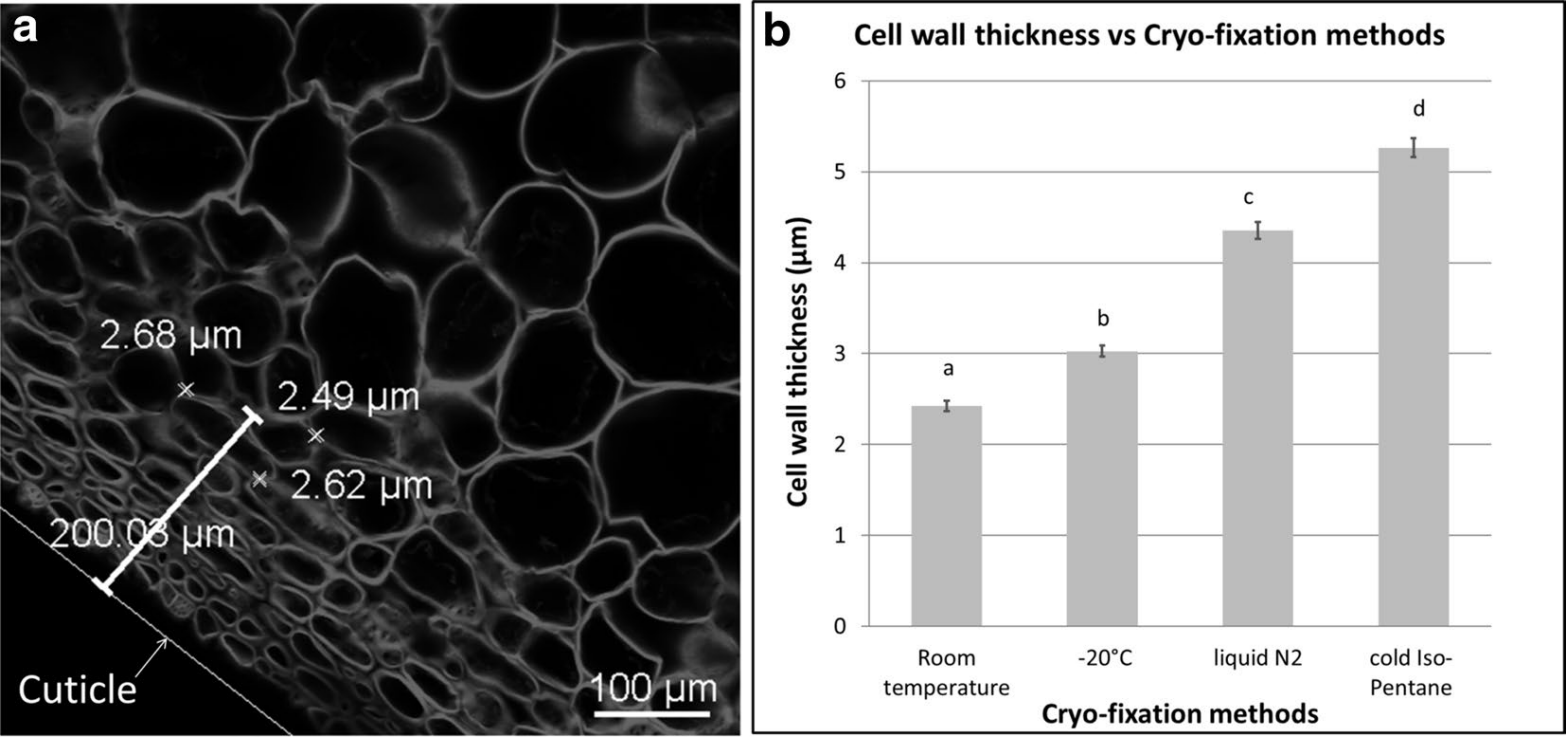
**a** Cell wall measurement on fresh apple tissue stained with Congo Red; **b** cell wall thickness according to different cryo-fixation methods. Bars: standard error of the mean (n = 80 for each condition)

The freezing process impacts turgid tissue structure due to ice crystal formation. The different cryo-fixation methods of AO infused samples were compared with regard to the apparent integrity of cell walls (Fig. 2). Observations at approximately − 25 °C revealed that slow freezing at − 20 °C (Fig. 2a, b), fast freezing in liquid nitrogen (Fig. 2c, d), and fast freezing in cold isopentane (Fig. 2e, f) impacted the overall tissue structure from most to least. These results agree with the impact of the rate of ice nucleation: the faster the freezing technique is, the less ice nucleation is present, and the better that structures are preserved. Slow-freezing under microwaves was also tested as an alternative to fast-freezing. Applying low-power microwaves during freezing drastically reduced the growth of ice nuclei and limited cell damage due to ice expansion [18]. The fruit tissue cryo-fixed by this technique yielded remarkable results with regard to the preservation of its cell integrity (Fig. 2g, h). This was particularly the case for the grape berry, for which tissue integrity was the most difficult to preserve. However, this promising technique still needs development to optimize its parameters, such as microwave power, freezing temperature and processing time. The preservation of inner cells in apple and grape tissues was more efficient using cooled isopentane freezing (Fig. 2e, f) than using microwave freezing (Fig. 2g, h). This may be explained by the short processing time of isopentane freezing (60 s) compared to that of 3 h for the microwave technique due to the air blast freezer used. Furthermore, by avoiding the Leidenfrost effect, isopentane freezing was preferred over liquid nitrogen freezing. However, the measurement of cell wall thickness as a function of freezing conditions revealed an impact of the cooling rate on cell wall thickness (Fig. 3). Congo Red-stained apple cell walls at approximately 200 µm from the cuticle were 2.5-fold thicker in tissue frozen by cold isopentane than in fresh tissue (room temperature), while tissue frozen at − 20 °C or by liquid nitrogen gave an intermediate average thickness.

### Observation of diffusible compounds

**Fig. 4:**
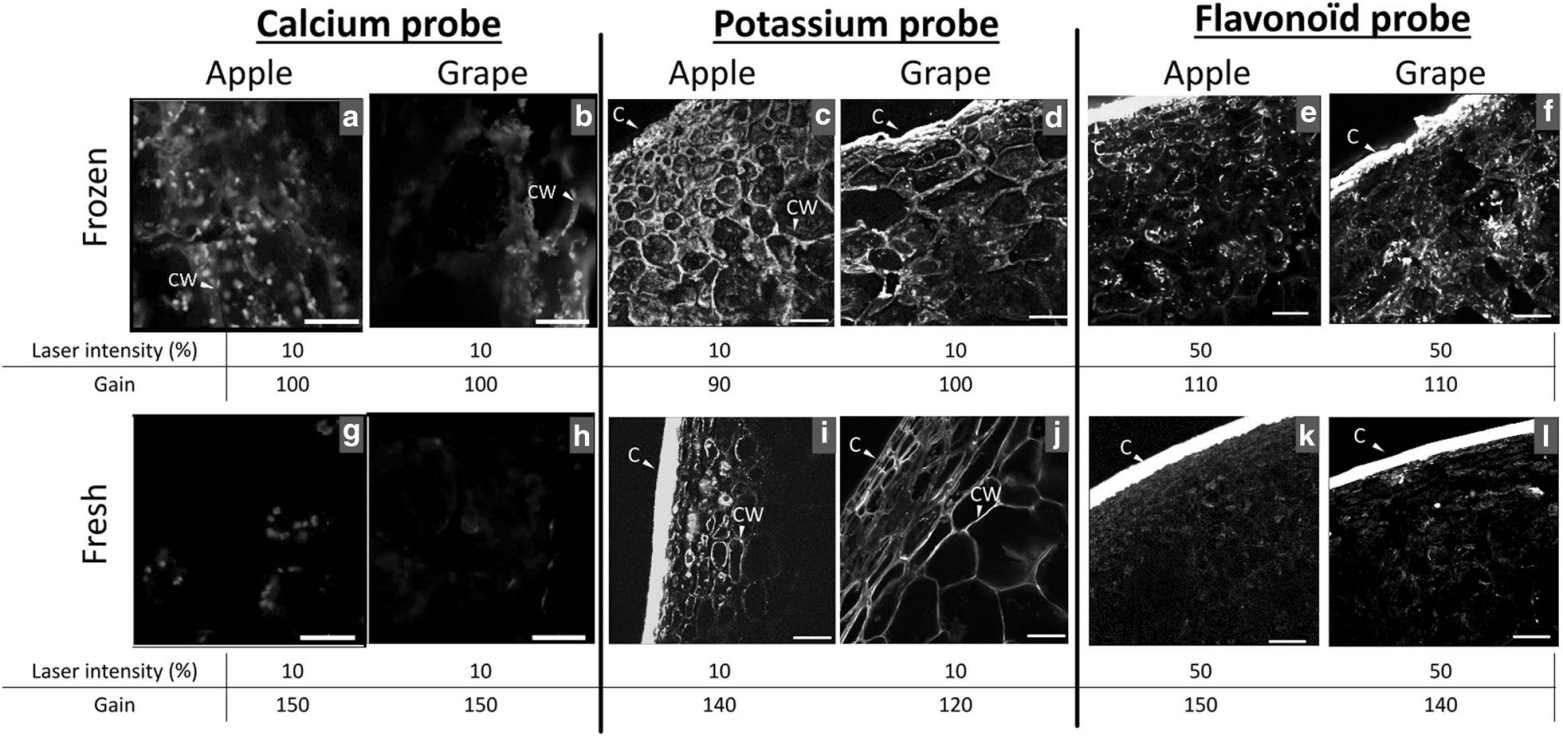
Cryo-observation of apple **a**, **c**, **e** and grape **b**, **d**, **f** tissues infused by Fluo-3 (calcium), PBFI (potassium), DPBA (flavonoid) dyes and cryo-fixed in cooled isopentane. Fluorescence observations in fresh apple fruit (**g**, **i**, **k**) and grape berry (**h**, **j**, **l**) sections directly stained by Fluo-3, PBFI and DPBA. C: Cuticle, CW: Cell Wall. Scale bar: 50 µm for calcium probe and 100 µm for potassium and flavonoid probes. Laser excitation and gain are indicated to stress the difference in fluorescence intensity recovered between fresh and frozen samples. The applied fluorescence intensity of the cuticle is due in part to autofluorescence related to the presence of phenolic compounds

As an application of the entire process on turgid plant organs, calcium, potassium and flavonoid compounds were localized in apple fruit and table grape berry tissue. Samples were independently infused with the calcium probe Fluo-3, potassium probe PBFI, and flavonoid probe DPBA. The samples were then fast-frozen in cold isopentane, cryo-sectioned and observed by cryo-LCSM (Fig. 4a–f). The results showed diffuse calcium staining in the cell wall, while fluorescence spots were observed within cells (Fig. 4a, b). Potassium and flavonoids were distributed in the entire fruit tissue (Fig. 4c–f), and specific locations appeared in the cytosol next to the cell walls (Fig. 4e, f). As a comparison, the direct staining of fresh sections yielded weaker labelling mainly due to the absence of intracellular staining (Fig. 4g–l). These observations may be attributed to low compound concentrations resulting from their diffusion during staining and loss during washes as well as from the higher diffusion of quenchers inducing faster fluorescence bleaching at room temperature [19, 20].

## Discussion

The experimental setup presented here was developed to image metallic cations and phenolic compound distributions in in vivo-like fleshy fruit by fluorescence confocal microscopy. Particular care was paid to the issues related to the low concentration and highly diffusible properties of these components. The fruit region of interest was established at the epidermal areas of grape and apple fruit. To allow full cell observation (range of cell diameter i.e., 10–100 µm) [21], section thicknesses needed to be adapted according to the tissue. Despite the particular physicochemical and mechanical characteristics of samples, sections with thicknesses of 100 µm were achieved but required dexterity for their handling, particularly for grape tissue. In addition, the physical state of the sample is essential when studying diffusible components [22]. For turgid plant organs such as fleshy fruit, water, which amounts to 80–85% of their weight, determines the morphological, physiological and physical properties of tissues at room temperature or in frozen solid states. Cryo-fixation limits the diffusion and redistribution of highly diffusible components [22, 23] but has several drawbacks. First, it requires that the sample be stained before freezing, and second, it faces the issue of structural damage by ice crystals [24, 25]. We found that fresh tissue infusion remains the most efficient and user-friendly method. It is well adapted to turgid plant tissue due to its porosity and exchange properties. Applied to apple and grape tissues, labelling was observed up to the fifth or sixth cell layer from the exposed area (approximately 100 µm for each layer) after two hours of staining. To reduce tissue destruction by ice crystals, cryogenic techniques have been developed. Plunge freezing [26], jet freezing, or slam freezing in cryogenic fluid or high pressure freezing (HPF) transform liquid water to a vitreous solid phase [27]. Currently, HPF is recognized as the method of choice for cryopreservation and is well adapted for ultramicrotome sections. This fast freezing process (≈ 0.5 ms) allows cell preservation up to 600 μm in thickness [28] and is optimal for investigating small cells and objects by the cryo-electron microscopy of vitreous samples (CEMOVIS) [29], cryo-correlative light transmission electron microscopy (cryo-CLEM) [30] or cryo-correlative light scanning electron microscopy [31]. These techniques are suited for preparing samples for cryo-transmission electron microscopy (cryo-TEM), which has a field of view of smaller than 100 µm^2^ and is thus not suitable for large sample sizes when a field of view of more than 500 µm^2^ is required. In that context, hand freezing at atmospheric pressure remains the simplest, fastest and most repeatable method of cryo-fixation, as samples are dipped in a cryogen, such as cold isopentane. The hand freezing method used here was inspired by the freezing step involved in the Tokuyasu method, with no sucrose infiltration, as fruit tissues are naturally rich in osmolytes that logically act as natural cryoprotectants [32]. Cryo-observation in light microscopy has already been developed [12], but to our knowledge, only a few commercial or homemade fluorescence microscopes equipped with a cryo-chamber cooled by liquid nitrogen are available. They are specifically used to observe vitrified ultrathin sections by fluorescence microscopy as a preliminary step prior to observation by cryo-TEM. In available cryo-fluorescence microscopy setups, dry lenses with relatively long working distances (WDs) and limited numerical apertures (NAs) are used [30]. Recently, a prototype of a LSCM stage and objective lens were described for the high-resolution cryo-observation of sub-cellular localization of animal proteins [20]. In our setup, the inverted optic of the LSCM possesses a short WD that maximizes the NA. These are key factors determining the fluorescence sensitivity and spatial resolution required to localize compounds at low concentrations, such as metallic cations or phenolic compounds in fruit tissue, with a large field of view.

The freezing of water-rich biological materials has been reported to affect cell contraction and the swelling of cell walls [33]. In the present study, the cell wall thickness was observed to vary according to the apple cryo-fixation temperature. As postulated [33], the extracellular water medium freezes first and provokes an outward water flux from the cells to osmotically equilibrate intracellular and extracellular media. Water in biomaterials is also known to expand by almost 9% during freezing and develops transient stresses when the material is frozen from all sides [34]. The cell wall swelling observed in relation to the freezing rate and temperature may be related to these mechanisms, but further studies are required to understand the water behaviour in the fruit cell wall during cryo-fixation. Although water flux and osmolyte redistribution may have occurred during sample cryo-fixation, which impacted the diffusible component distribution, these artefacts may have been limited. Indeed, the two types of calcium labelling distribution observed in both apple and grape tissues showing diffuse staining in cell walls and intense fluorescence spots in cells (Fig. 4a, b) were in agreement with their reported presence in nuclei, vesicles and plant cell walls [22, 35]. Furthermore, the observation of potassium labelling in the apoplast and cell wall of fresh tissue (Fig. 4i, j) and in cells (Fig. 4c, d) of frozen tissue was in agreement with its apoplastic, cytosolic and vacuolar location in fleshy fruit cells [36]. In the literature, calcium and potassium were measured directly on isolated cells and organelles using a fluorescent probe. Lastly, flavonoid distribution mainly observed in the cytosol close to the cell wall supports their putative sites of synthesis. Conversely, their proposed accumulation in the vacuole was not dominant in the present observations in apple and grape, but such localization remains a matter of debate (Fig. 4e, f) [37]. The cellular localization of these different diffusible compounds in the two fleshy fruit parenchymal tissues demonstrated the benefit of the cryo-method.

## Conclusion

The localization of highly diffusible and low concentrated components such as metallic cations and flavonoids was achieved in fluorescence mode by LSCM. The reduction of component mobility was realized by keeping samples in a frozen state during the entire preparation process and by the design and adaptation of a cryo-LSCM setup for the observation of frozen sections. The successful localization of calcium, potassium and flavonoids in apple and grape fruits as a model of turgid tissue illustrated the benefits of the cryo-approach. The results indicated that the freezing temperature and cooling rate remain key parameters in the preservation of hydrated tissue integrity and require further studies to better control the associated osmotic-related structural rearrangements. The extension of this approach to other highly diffusible compounds will benefit from the development of specific and sensitive diffusible fluorescent markers. This approach opens new opportunities for studies of small metabolites and ions in the plant sciences.

## Methods

### Plant material

**Fig. 5:**
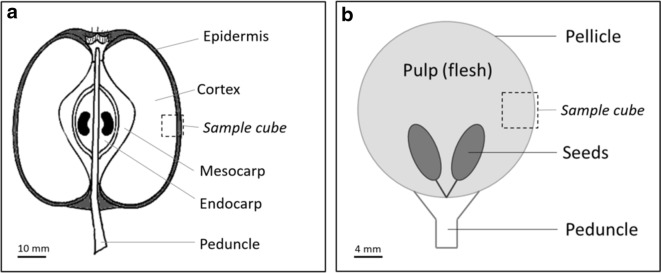
Apple A) and grape berry B) sampling and nomenclature

The fruit tissue nomenclature used in this study is shown in Fig. 5. Gala apples and Italia white table grapes were obtained from a local retail store. Regions of interest corresponding to the apple cortex and grape berry with epidermis were sampled as cubes of approximately 0.125 cm^3^ using a razor blade (Fig. 1).

### Sample preparation for microscopy

Samples were stained by infusion or perfusion before freezing according to different paths followed by cryo-sectioning and cryo-observation. For comparison, samples were also stained by a conventional method and observed in an unfrozen state.

#### Staining

- *Fluorescence stains:* Acridine orange (AO hydrochloride salt; MERCK Calbiochem, France) was prepared as a *0.02% w/v solution in 0.01* *M PBS buffer (pH 7)*. Congo Red stain (Congo Red powder, FLUKA, Switzerland) was prepared as a 0.1 mg/mL solution in deionized water at pH 5. Fluo-3 calcium probe (Fluo-3 pentapotassium salt, Thermo Fisher Scientific, France) and PBFI potassium probe (PBFI, tetraammonium salt, Thermo Fisher Scientific, France) were prepared as a 0.1 mg/mL solution in MES (25 mM) + Tris (10 mM) buffer (pH 6.0) [17] and kept as a stock solution in an amber flask at 4 °C. The phenolic probe DPBA (2-Aminoethyl diphenylborinate, Sigma-Aldrich, UK) was prepared by dissolving 20 mg in 5 mL of ethanol and 15 ml of phosphate buffer solution 0.01 M (pH 7).
- The conventional staining of fruit sections (see below for sectioning details) was performed by applying 2 mL of staining solution onto the sections for 5–10 min at room temperature. Sections were then rinsed 3 times with buffer for approximately 5 min each.
- Staining by infusion of fruit samples: Cubes of apple and grape were bathed in staining solutions for 2 h at 5 °C (Fig. 1a).
- Staining by perfusion of entire fruit: Staining solution was introduced in specific areas of the entire fruit using a syringe (5 mL) filled with 2 mL of staining solution connected to 10 cm of capillary tubing (inner diam. 1 mm) inserted in the fruit. The tubing was perforated at the contact zone in the fruit, with a 10-mm length and a 0.8-mm pore size (approximately 10 apertures) to allow for the diffusion of the stain. To ensure the flow of the stain in the fruit, the syringe outlet was set 10 cm above the capillary outlet. The flow rate was approximately 125 µL/h. Diffusion was applied overnight at 5 °C (Fig. 1b).

#### Cryo-fixation

Cryo-fixation was achieved following several methods to obtain different cooling rates:

- The slow freezing of samples at − 20 °C was conducted for at least 24 h in a conventional freezer.
- Two fast-freezing methods were tested using the cryogen: for the first one, the sample was directly plunged in liquid nitrogen, whereas for the second one, it was plunged in isopentane (2-methylbutan anhydrous > 99%, SIGMA) cooled by liquid nitrogen. In both cases, the freezing duration was 60 s.
- Freezing under microwave [38] was conducted with the following parameters: microwave equipment (SAIREM, France) operated at a frequency of 2450 MHz, the microwave chamber stabilization time was 30 min at 5 °C, a temperature of − 30 °C was set using an air blaster (ACFRI, France), the microwave power was 5 ± 0.1 W, and the duration of freezing was 3 h.

#### Sectioning

- Fresh specimens were sectioned at room temperature using a vibrating blade microtome (Vibratome HM 650 V, MICROM, France), stained by a conventional method and collected between 60 × 24 mm glass cover slips (#1) (Thermo Fisher Scientific, Germany) separated by a 250-µm-thick spacer (Gene Frame, 25 µL, 1 × 1 cm^2^, Thermo Fisher Scientific, UK). The sectioning parameters used were a section frequency of 60 Hz, a vibration amplitude of 1.0 mm, and a cutting speed of 1.0 mm/sec. Sections were cut to a thickness of 100 µm.
- Frozen specimens were cryo-sectioned using a cryotome (microtome cryostat HM 500 OM, MICROM, France) operated at − 20 °C. The sample cube was fixed on the support section using a water droplet free of cryo-protectant. The cryotome steel blade was a type C profile (16 cm length). The cutting speed was fixed at 1 mm/sec, and the thickness of the sections was 100 µm. Sections were picked up and placed between two 22 × 22 mm #1 glass cover slips (Thermo Fisher Scientific, Germany), which were sealed by frozen water microdroplets.

In all cases, sectioning was performed on the stained sample contact area.

The transfer of cold sections within cover slips to the confocal microscope was conducted rapidly over liquid nitrogen.

### Laser scanning confocal microscopy (LSCM) observations

LSCM (Eclipse Ti inverted microscope, NIKON Inc. Japan) was used both in bright field and fluorescence modes. Observations were made using a 20 × magnification objective lens and, if needed, a numerical zoom of 3x. The laser excitation and fluorescence emission wavelengths were 488 nm for acridine orange, 500–530 nm for Fluo-3 and DPBA dyes, 488 nm and 600 nm for Congo Red dye, and 375 nm and 500–530 nm for PBFI dye. Laser intensity and gain were adjusted visually for sample fluorescence.

### Cryo-observation by LSCM

**Fig. 6:**
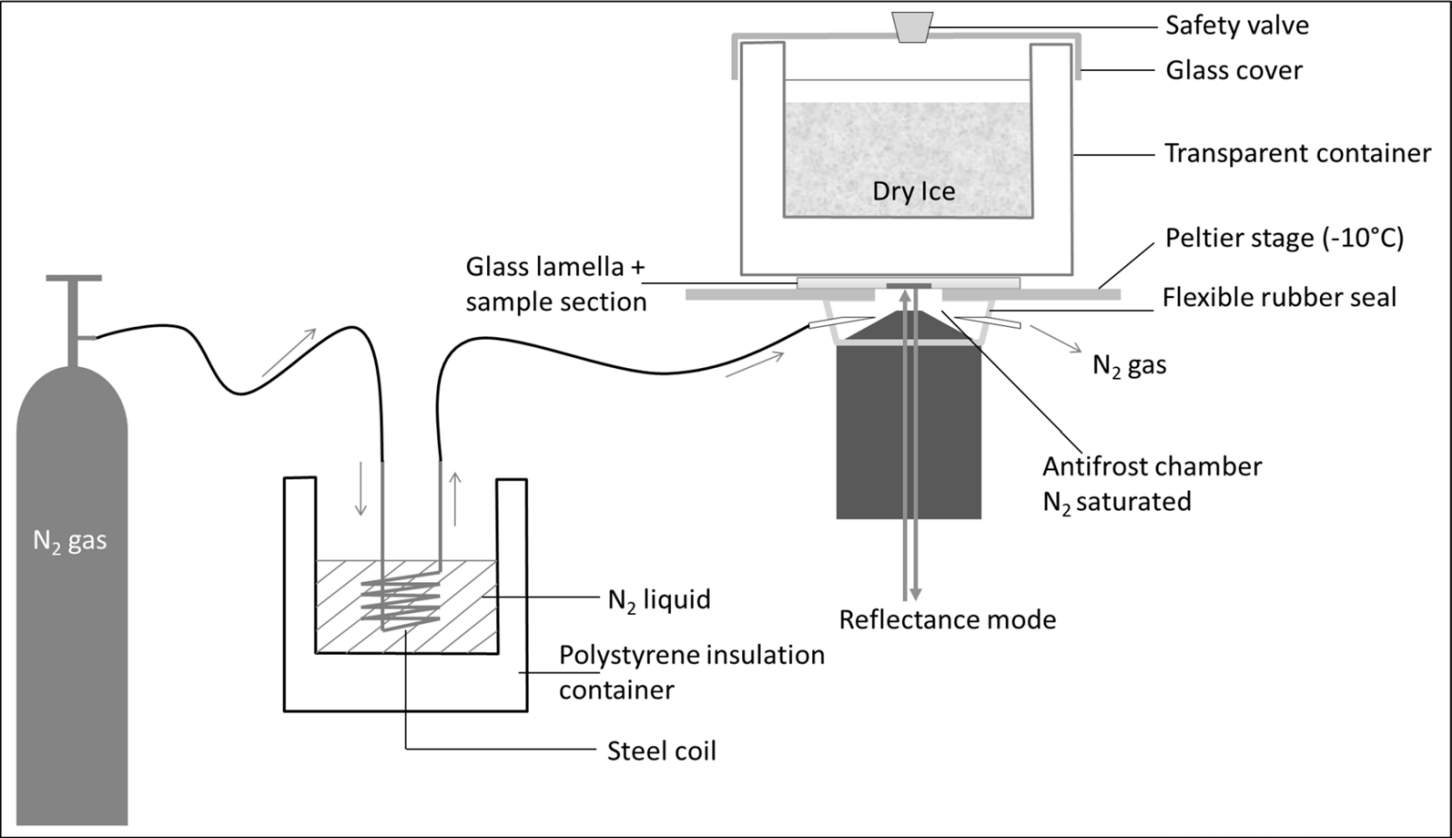
Setup for LSCM cryo-observation

The critical points to manage for LSCM cryo-observation were sample section warming induced by environmental factors and by the laser during observation as well as frost formation on the sample slide. The sample slide was placed onto a Peltier stage (PE100, Linkam Scientific, Epsom UK) under which an adaptable flexible rubber seal was fixed between the objective lens and the stage (Fig. 6). This closed space between the lens and the stage was flushed by a cooled nitrogen gas flux to prevent frost formation on the sample slide. A glass container filled with dry ice was placed on top of the sample slide. The measured temperature of the Peltier stage was approximately − 25 °C. The container transparency allowed the microscope optical condenser to observe the sample in a bright field.

### Cell wall thickness measurement

Cell wall thickness was specifically evaluated on four apple fruit samples after staining with Congo Red (a cell wall specific dye) and observed at room temperature after slow freezing at − 20 °C and after fast freezing by liquid nitrogen and by isopentane. Four sections per sample were recovered, and five cell wall measurements (a total of 80 measurements) were performed on the cell layer at approximately 200 µm from the cuticle using NIS Analysis software (Nikon).
